# Monoclonal Antibodies and Invasive Aspergillosis: Diagnostic and Therapeutic Perspectives

**DOI:** 10.3390/ijms23105563

**Published:** 2022-05-16

**Authors:** Xihua Lian, Amy Scott-Thomas, John G. Lewis, Madhav Bhatia, Sean A. MacPherson, Yiming Zeng, Stephen T. Chambers

**Affiliations:** 1 Department of Pathology and Biomedical Science, University of Otago, Christchurch 8140, New Zealand; xihua.lian@postgrad.otago.ac.nz (X.L.); amy.scott-thomas@otago.ac.nz (A.S.-T.); john.lewis@otago.ac.nz (J.G.L.); madhav.bhatia@otago.ac.nz (M.B.); sean.macpherson@cdhb.health.nz (S.A.M.); 2 Department of Medical Imaging, The Second Clinical Medical School of Fujian Medical University, Quanzhou 362000, China; 3 Steroid and Immunobiochemistry Laboratory, Canterbury Health Laboratories, Christchurch 8140, New Zealand; 4 Haematology Department, Christchurch Hospital, Christchurch 8011, New Zealand; 5 Department of Internal Medicine (Pulmonary and Critical Care Medicine), The Second Clinical Medical School of Fujian Medical University, Quanzhou 362000, China; zeng_yi_ming@126.com

**Keywords:** monoclonal antibody, invasive aspergillosis, *Aspergillus* infection, diagnosis, therapy

## Abstract

Invasive aspergillosis (IA) is a life-threatening fungal disease that causes high morbidity and mortality in immunosuppressed patients. Early and accurate diagnosis and treatment of IA remain challenging. Given the broad range of non-specific clinical symptoms and the shortcomings of current diagnostic techniques, most patients are either diagnosed as “possible” or “probable” cases but not “proven”. Moreover, because of the lack of sensitive and specific tests, many high-risk patients receive an empirical therapy or a prolonged treatment of high-priced antifungal agents, leading to unnecessary adverse effects and a high risk of drug resistance. More precise diagnostic techniques alongside a targeted antifungal treatment are fundamental requirements for reducing the morbidity and mortality of IA. Monoclonal antibodies (mAbs) with high specificity in targeting the corresponding antigen(s) may have the potential to improve diagnostic tests and form the basis for novel IA treatments. This review summarizes the up-to-date application of mAb-based approaches in assisting IA diagnosis and therapy.

## 1. Introduction

Invasive aspergillosis (IA), the most devastating form of *Aspergillus* infection, refers to an opportunistic, life-threatening, acute and rapidly progressing infectious disease [1]. IA is predominately caused by *Aspergillus fumigatus*, which is ubiquitous within our environment. Other *Aspergillus* species such as *Aspergillus flavus*, *Aspergillus niger*, *Aspergillus terreus* and *Aspergillus nidulans* can also cause IA [2]. IA usually occurs in immunosuppressed patients, including neutropenic hosts, patients undergoing prolonged treatment with corticosteroids, and allogeneic hematopoietic stem cell transplant (HSCT) and solid organ transplant (SOT) recipients [3,4]. With delayed diagnosis, the mortality of IA in immunodeficient hosts can be high as 90%.

The increasing number of immunodeficient patients due to immune suppressive therapy accounts for the growth in IA numbers. Globally, IA causes in excess of 200,000 mortal infections every year [5,6]. Additionally, patients suffering from IA have an increased risk of complications from viral infections such as influenza and COVID-19 [7,8]. IA cases secondary to viral infections have complex clinical presentations leading to further difficulty and delays in diagnosis and therapy [9,10]. Accurate diagnosis and a targeted antifungal treatment are the fundamental requirements for reducing both the morbidity and mortality of IA.

Monoclonal antibodies (mAbs), produced by a single B-lymphocyte clone, have high specificity in targeting the corresponding antigen(s) and have the potential to improve diagnostic tests, forming the basis for novel IA treatments. MAbs have been developed for diagnosis and therapy of cancers [11,12], autoimmune disease [13], asthma [14] and infectious diseases [15,16] including COVID-19 [17,18] but have yet to reach their full potential for improving the outcomes of IA. This review examines the potential usefulness of mAbs in diagnosing and treating IA from different perspectives.

## 2. Pathogenesis of Invasive Aspergillosis

The conidia of the *Aspergillus* species that causes IA are small (approximately 2.5–3.5 μm in diameter), and covered in a hydrophobic layer. They are very robust under normal atmospheric conditions, and can remain airborne, dispersing widely on air currents after release [19], and can be inhaled unless removed by physical filtration [20,21]. Healthy hosts clear inhaled conidia that become trapped in the mucociliary escalator effectively, and the immunological cellular defences clear those that penetrate as far as the alveoli [21,22,23]. In contrast, the conidia can escape clearance from the respiratory tract in immunodeficient patients.

After inhalation, the hydrophobic exterior protein cover and melanin protect the conidia from being recognized and attacked by the host by masking of the pathogen-associated molecular patterns (PAMPs) [21,24,25,26]. Conidial constituents that mediate the adherence and colonization to the host epithelial cells include conidial sialic acid residues [27,28] and fucose-specific lectin A [29]. Subsequently, surviving conidia start to swell and release surface hydrophobin and melanin, exposing PAMPs that interact with pattern recognition receptors (PRRs) on pulmonary epithelial cells. Conidial wall β (1-3)-glucan is recognized by dectin-1 and initiates engulfment by epithelia [30]. This internalization process is promoted by the interaction of conidial calcineurin A (Cal A) and integrin α5β1 [31] and the activation of cellular molecules [32,33,34]. Engulfment of conidia induces the inflammatory response in the epithelia. Most conidia are killed during this offensive response; however, the conidia that escape phagocytosis continue to swell and germinate into filamentous hyphae. Galactosaminogalactan (GAG), a soluble molecule secreted by the hyphae, mediates the fungal attachment to the host pulmonary epithelial cells [35,36]. In addition, GAG production around the hyphae leads to the reduction of β (1-3)-glucan exposure. This blocks the interaction between the β (1-3)-glucan and the dectin-1, which attenuates the inflammatory responses and fungal death [35]. GAG also has been shown to induce neutrophil apoptosis, inhibit the formation of neutrophil extracellular traps (NETs) termed NETosis and pre-inflammation, so as to protect the *Aspergillus* from killing [23,37,38].

Hyphae invading host tissues grow quickly with an extension rate of 20.2 to 25.4 μm/h [39], and release proteases such as cysteine and serine protease, metalloproteinase, and elastase that degrade the host epithelial tissue and generate nutrients that support further fungal growth [40,41]. *Aspergillus* hyphae express and secrete multiple mycotoxins that aggravate the damage to host pulmonary cells and basement membrane [42] or affect the host immunity [43]. Of these, gliotoxin facilitates the internalization of *Aspergillus* conidia by type II human pneumocyte cells [33] and prohibits the host’s immune response by inducing apoptosis in macrophages and monocytes [43,44,45]. Fumagillin is cytotoxic to lymphocytes, leading to cellular immune suppression [46,47]. It also causes destruction of the pulmonary tissue and promotes fungal growth [48]. These virulence factors have the dual properties of disrupting the host’s immune system and facilitating fungal growth, leading to hyphae crossing endothelial barriers and invading blood vessels. This process may lead to rapid dissemination and colonization of remote tissues and organs, resulting in the histologically typical features of IA lesions that are characterised by tissue injury, necrosis and hypoxia [22].

**Figure 1:**
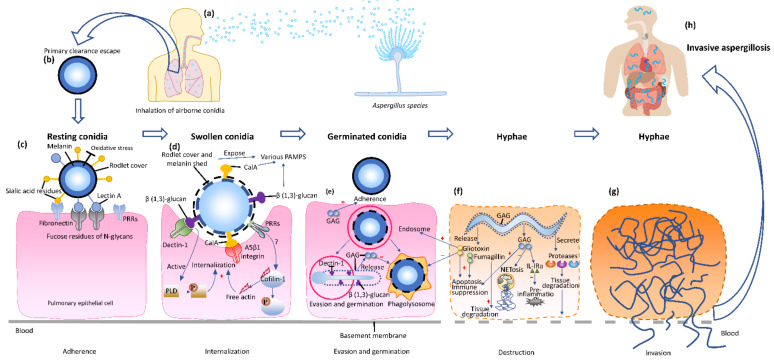
The pathogenesis of invasive aspergillosis. (**a**) Inhalation. Airborne conidia are inhaled into the respiratory system of the immunosuppressed host. (**b**) Clearance escape. The resting conidia escape primary clearance and immune attack. (**c**) Adherence. The rodlet protein cover and melanin of the resting conidia mask the pathogen-associated molecular patterns (PAMPs) and protect the conidia from oxidative stress and the environment (UV) and host (reactive oxygen species, ROS), and from being recognized and attacked by the host. Lectin A and sialic acid residues mediate the adherence and colonization onto the fucose residues of N-glycans and fibronectin of the pulmonary epithelial cells. (**d**) Internalization. The swollen conidia release the surface hydrophobic layer and melanin, exposing more PAMPs, such as β (1-3)-glucan and calcineurin A (Cal A), to be recognized by dectin-1 and integrin α5β1, respectively, on the epithelial cell wall, inducing the internalization. In addition, the formation of actin polymerization and activation of phospholipase D (PLD) both boost the internalization. (**e**) Evasion and germination. The swollen conidia in the endosome evade the phagolysosome killing and germinate into tubes and hyphae. Galactosaminogalactan (GAG) on the hyphae wall can be released as a soluble molecule, mediating adhesion, inhibiting phagocytosis and suppressing the host inflammatory responses by masking of β (1,3)-glucan on the hyphal wall. (**f**) Destruction. GAG released by mature hyphae facilitates the induction of neutrophil apoptosis and prohibits formation of neutrophil extracellular traps (NETosis), and pre-inflammation by induction of IL-IRa. The secreted gliotoxin and fumagillin are involved in pathogen internalization, cell apoptosis, host immune inhibition and tissue degradation. (**g**) Invasive aspergillosis. The tissue-invasive hyphae penetrate the alveolar epithelia and basement membrane into the blood vessel, disseminating to the whole body and causing IA (**h**). +: promote; −: inhibit.

In summary, airborne *Aspergillus* conidia reach the airways by inhalation, but hyphae are the invasive fungal form in human infections. *Aspergillus* cell wall components facilitate the adherence and induce endocytosis by host cells. The proteases released by *Aspergillus* hyphae promote the remote invasion of the parenchyma and blood vessels. Various toxins co-impede the immune system function of the individuals and aggravate the severity of IA (Figure 1).

## 3. Monoclonal Antibodies and Diagnosis of Invasive Aspergillosis

### 3.1. Challenges of Invasive Aspergillosis Diagnosis

The early and accurate diagnosis of IA is a major challenge because there are a broad range of non-specific clinical symptoms, such as cough, fever, chills, dyspnea, hemoptysis, chest pains, headaches, and weight loss associated with IA [21,49]. A full range of diagnostic methods have been evaluated for IA (Table 1). The current consensus, from the European Organization for Research and Treatment of Cancer and the Mycoses Study Group Education and Research Consortium (EORTC/MSGERC) [50], establishes the identification of *Aspergillus* in biopsy tissue samples, or a positive culture of pathogens from a specimen taken from a normally sterile site as the gold standard for diagnosis of IA. In immunosuppressed patients, particularly those with neutropenia and thrombocytopenia, the invasive procedures needed to obtain tissue samples are hazardous, thereby limiting their availability [21,51]. Consequently, most patients are either diagnosed as “possible” or “probable” cases but not “proven” because of the shortcomings of the diagnostic techniques currently available [50,52,53]. The criteria for a “probable” IA diagnosis require the presence of a host factor, which is usually immune suppression, clinical features such as fever unresponsive to antibacterial treatment, and mycologic detection. Those who meet the criteria for a host factor and a clinical characteristic but without mycological evidence should be considered as “possible” IA cases.

### 3.2. Monoclonal Antibody-Based Diagnostic Approaches for Invasive Aspergillosis

The difficulty in diagnosing IA has spurred many investigators to explore the value of both in vitro and in vivo approaches using mAbs. In recent decades, these methods have gradually been adopted clinically. The *Aspergillus* cell wall components, such as polysaccharides and glycoproteins, and secreted functional molecules could all be potential targets for assays using platforms based on mAbs to indicate infection with *Aspergillus* species. All of these techniques take advantage of the highly specific interaction with the target antigen and mAb.

#### 3.2.1. The Detection of *Aspergillus* Antigens in Blood and Urine Using Monoclonal Antibodies

Soluble molecules released or metabolized by *Aspergillus*, such as galactomannan (GM) and other galactomannoprotein [54,55,56] in the blood and bronchoalveolar lavage fluid (BALF), are promising biomarkers for *Aspergillus* infection. These soluble antigens in various specimens can be detected by the mAb in vitro and some of the assays, such as lateral flow device (LFD) analysis, have been designed as a “point-of-care (POC)” diagnostic method [57,58,59]. The detection of these circulating or break down antigens provides evidence of *Aspergillus* infection in the early infection period and may prompt the initiation of antifungal treatment but does not identify the infecting species. 

The most common in vitro diagnostic approach is the mAb-based antigen detection sandwich ELISA that is used to detect the *Aspergillus* antigens in blood samples. The mAb EB-A2 against GM binds to an epitope of β-(1-5)-connected galactofuranose residues, and is the first antibody to have been widely investigated in an ELISA [54]. The Platelia™ *Aspergillus* EIA, an mAb EB-A2-based GM detection sandwich microplate assay, is a well-established commercial test that may detect GM at an early stage of infection [60,61], supporting a diagnosis of IA. The sensitivity of this GM assay in serum ranges from 41% to 78%, which is lower than that of BALF, which varies from 60% to 100%. The specificity of this assay in serum is 60% to 95%, which is again lower than the 85% to 98% tested in BALF [62,63,64]. The sensitivity of this GM assay is further reduced by earlier treatment with antifungal agents [50,65]. 

In an effort to improve the performance of a biomarker assay for *Aspergillus* antigens, a variety of other assays based on novel mAbs have been investigated. Hao et al. [66] produced 17 candidate antibodies and developed an assay using two of these in sandwich ELISA to detect *Aspergillus* antigens in a rabbit model of IA. Wang et al. [67] have developed a sandwich ELISA using mAb against recombinant Afmp1p and Afmp4p that detects the antigen in culture filtrate and serum from *Aspergillus* infected rabbit. Ansari and colleagues recently reported two mAbs that recognised recombinant *A. flavus* mannoprotein 1 (Aflmp1), that were both highly specific to *A. flavus* and *A. parasiticus* cell wall fragments [68]. Our team has produced two mAbs including 1D2 and 4E4 against *Aspergillus* cell wall glycoproteins, which are capable of testing the secreted antigens in culture media and plasma antigens in *A. fumigatus* infected IA mice [69]. These studies display promise in detecting various circulating antigens in pre-clinical research, supporting development for clinical application later. 

An LFD has been developed that detects antigens in plasma and BALF samples. This is a non-culture mAb-based immunoassay that can be used at the POC to detect *Aspergillus* antigens of human IA [58,70]. The primary LFD test is a JF-5 based assay, which can detect (galacto)mannoprotein antigens in different samples [71]. In BALF specimens, the sensitivity of LFD testing varies from 58% to 89%, and the specificity ranges between 68% and 100% [58,72,73,74,75] but is less sensitive on plasma. Further studies are needed to determine the clinical utility of this test.

There has been interest in using urine as a diagnostic sample in LFD, as it can be readily obtained and may be useful for monitoring patients at home. Marr and colleagues reported that an mAb476-based LFD test using urine can be beneficial in IA diagnosis in high-risk hosts [76]. MAbs, such as EB-A2 and WF-AF-1, have been well standardised using the LFD technique and are now available commercially [58,77]. This test has been reported to be of value in the diagnosis of COVID-19-associated pulmonary aspergillosis [8]. Given that the LFD urine test is a rapid, non-invasive, economic, convenient, and easy to perform by untrained personnel, it may be used as a “home-testing” technique to monitor the disease progress and treatment efficacy [57,78]. The combination of LFD with other methods such as PCR may increase the diagnostic accuracy significantly [73,79].

#### 3.2.2. The Use of *Aspergillus* Monoclonal Antibodies for Microscopy and Imaging

- Microscopy

MAbs have also been used as probes to detect *Aspergillus* in formalin-fixed and paraffin-embedded tissue specimens using immunohistochemistry (IHC) or immunofluorescence (IF) to identify the bound complexes [69,80].

An early study indicated that an mAb-based IHC procedure was able to differentiate *Aspergillus* species from other filamentous fungi and assisted the clinical diagnosis and treatment of IA [81]. In human formalin-fixed and paraffin-embedded lung, liver and skin tissues, mAb EB-A2 was able to identify *Aspergillus* hyphae and fungal fragments within phagocytic cells [82,83]. Another commercial mAb, WF-AF-1, has been employed to detect *Aspergillus* antigens in specimens embedded in paraffin blocks from pediatric patients [80]. Tests on specimens offer a rapid identification of *Aspergillus* elements, particularly in those patients whose microbiological cultures are negative. Other non-commercial mAbs specific to *Aspergillus* have been used for detecting *Aspergillus* species in infected animal tissue samples and may have future clinical applications [66,69]. 

This technique may identify *Aspergillus* species more rapidly than traditional culture techniques and does not rely on the viability of the organism in samples from patients who have been heavily treated with antifungal agents. The antigens released or metabolised into the parenchymal tissue can also be detected by mAb-probed IHC/IF. This technique can also be used to identify pathogens in stored tissues to aid retrospective studies of fungal infection.

Recently, Amich et al. reported that three-dimensional light sheet fluorescence microscopy (3D-LSFM) could be used to visualize and quantify the location of fungal infection lesions ex vivo, as well as the immune reaction at the cellular level in whole extracted lungs from a mouse model [84]. The fluorescence labelled mAb JF5 (JF5-DyLight 655) was used to locate the *A. fumigatus* infection lesions. Henneberg et al. confirmed this result using DyLight-650-conjugated JF5 [85]. This novel ex vivo diagnostic tool adds an alternative mAb-based approach for IA diagnosis.

- Imaging

MAb-directed in vivo molecular imaging is a novel technique designed to identify specific *Aspergillus* species causing infective lesions in living animals. *Aspergillus* cell wall components identified by the tracer conjugated to mAb detected by PET/MR has been reported to be sufficiently sensitive to determine the infection site at a very early stage in IA animal models during life [49,86].

Molecular imaging is defined as an optical method that is able to qualitatively and quantitatively display specific biological profiles at the tissue, cellular or molecular level via signals released by reporters or tracers (such as radionuclides, metal nanoparticles) conjugated with highly specific binding agents (such as mAb). Changes identified at the cellular and molecular level in early stage disease may become apparent before anatomical changes can be seen. To date, two key non-invasive molecular imaging modalities have been reported in animal models [87]. The first one is an optical imaging technique that relies on the photon production by either bioluminescent [88,89] or fluorescent [89,90] reporters of gene-modified pathogens [90,91]. The second is based on the mAb-conjugated radionuclide release of either positrons (e.g., positron emission tomography, PET) [92,93] or γ-rays (e.g., single-photon emission computed tomography, SPECT) [92]. The combination of these functional imaging modalities with conventional imaging (such as CT and MRI) produce high-contrast images of tissue structures capable of showing the specific site of lesions [49,86]. Molecular imaging is showing promise as a non-invasive, precise method for the early diagnosis of IA in vivo, facilitating a personalized therapeutic regimen and treatment follow-up [94] as well as reducing the unnecessary drug application and hospital expenditures.

- Bioluminescence Imaging

Bioluminescence imaging (BLI) relies on the detection of photons emitted during the biochemical generation of light by the luciferase enzyme catalyzing the luciferin oxidation [95]. The firefly luciferase gene regulated by the *gpdA* promoter has been successfully inserted into the genome of *A. fumigatus* [96] to locate the infection pathogens in BLI. In addition, BLI is capable of screening the infection progress over time in the same animal to define the time course of an infection, reducing the need for sacrificing multiple animals at various time points [96,97]. BLI can only detect the gene-modified *Aspergillus* strains and has limited depth penetration, but is sufficiently sensitive and specific to monitor the pathogenesis and treatment of IA both in vitro and in vivo in small animals [91,96,97,98,99]. Nevertheless, BLI is not applicable to studies in humans [96,97].

- Immune PET/MRI

Positron emission tomography (PET) scan is an imaging test that can help reveal both normal and abnormal metabolic activity or biochemical functions in the tissues and organs by radioactivity. Given that PET has limited accuracy in pinpointing the precise location of the disease, it is usually combined with CT or MRI and referred to as PET/CT or PET/MRI scanning. Radiolabelled mAb-mediated PET combined with MRI, termed immunoPET/MRI [100], is a comparatively new field in cancer diagnosis [101], but has not been fully explored as a tool in clinical IA diagnosis. The mouse mAb JF5 (mJF5) [102], which binds to antigens common to *A. fumigatus*, *A. flavus*, *A. niger* and *A. terreus*, binds to invasive hyphae rather than the resting conidia [87]. Rolle et al. [86] first reported a [^64^Cu] DOTA (1,4,7,10-tetraazacyclododecane-1,4,7,10-tetraacetic acid)-labelled mJF5-directed in vivo PET/MRI to determine the location of *A. fumigatus* in IPA mice, thereby diagnosing IPA specifically and non-invasively. This study indicated that radionuclide labelled-mJF5-mediated in vivo molecular imaging is capable of detecting *A. fumigatus* hyphae in mice and distinguishing *Aspergillus* infection from bacterial infection or sterile inflammation in lungs, emphasizing the promising nature of this technique for diagnosing IA. 

As a first step toward translating this diagnostic approach for possible clinical application, Davies and colleagues produced a novel humanized mAb JF5 (hJF5) specific for *A. fumigatus* antigens and conjugated it to [^64^Cu] DOTAGA (1,4,7,10-tetraazacyclododececane,1-(glutaric acid)-4,7,10-triacetic acid) or [^64^Cu] NODAGA (1,4,7-triazacyclononane,1-glutaric acid-4,7-acetic acid) [49]. This group found that the uptake of [^64^Cu] NODAGA-hJF5 in the lungs of *A. fumigatus* infected animals was significantly improved when compared to that in mice receiving [^64^Cu] DOTAGA-hJF5 and mJF5 tracers, demonstrating that the conjugation of ^64^Cu and the chelator NODAGA to hJF5 enhanced the performance of immunoPET/MRI in vivo for lung lesions. Furthermore, this radiolabelled antibody tracer can be used to monitor the treatment responsiveness of new antifungal regimens [49]. In addition to this, hJF5 mAb has been double-conjugated with both [^64^Cu] DOTAGA and DyLight650 and visualized by PET/MRI and 3D light sheet fluorescence microscopy (LSFM). This study gives evidence of the multiple applications of dual-labelled hJF5 for imaging, as it can be used to localize and quantify the *A. fumigatus* infection in vivo and ex vivo, as well as monitoring the responsiveness of antifungal azole treatment [85]. 

At present, all of these mAb-guided molecular imaging modalities have been applied only in pre-clinical IA studies, though they show promise in the diagnosis of IPA. Moreover, a multimodal imaging method is beneficial for in vivo evaluation of antifungal drugs in animal models [103]. Future work needs to be conducted to evaluate this innovative imaging method in IA animal models involving other organ infections, such as those of the brain, kidney and spleen, and to facilitate its translation to the clinical setting. 

## 4. Monoclonal Antibodies and Invasive Aspergillosis Therapy

### 4.1. Challenges of Invasive Aspergillosis Therapy

Effective anti-*Aspergillus* treatment closely depends on the initial treatment in the early course of the *Aspergillus* infection, recovery from neutropenia and, if possible, reversal of immune suppressive therapy. Because of the lack of sensitive and specific tests, many high-risk patients receive an empirical therapy regimen of high-priced antifungal agents before they are accurately diagnosed, or a prolonged treatment after proven diagnosis, posing a heavy economic burden on society and the patients’ families [104]. In addition, anti-fungal prophylaxis or improper drug usage may lead to antibiotic overuse, unnecessary adverse effects on the patient and resistance to *Aspergillus* species such as *A. fumigatus* [6]. 

As present, there are three main drug classes of antifungal agents: triazoles, amphotericin B and echinocandins [105,106,107,108]. All agents require long therapeutic cycles and have varying degrees of toxicity and various adverse effects. These include hepatotoxicity, neurotoxicity, impairment of renal function, nausea, vomiting, hypertriglyceridemia, and complex drug interactions with other treatment regimens. Furthermore, the mortality rate of IA in immunosuppressed individuals still ranges from 30% to 90% in spite of anti-*Aspergillus* therapy [5]. Surgery is another alternative for those patients who have limited lung lesions or for infections that do not respond to antifungal drugs. This has very limited application in those who are critically immunocompromised due to the high risk of fatal haemorrhage, death or other complications after surgery [21]. Because of these limitations, the development of new, rapid acting, effective and specific anti-mold therapeutic options is needed to manage the increasing population of immunosuppressed patients. MAb-based immunotherapeutic strategies may offer an avenue to develop such novel treatments because of the high specificity of mAb binding to the corresponding antigen.

### 4.2. Anti Aspergillus Activity of Monoclonal Antibodies

**Figure 2:**
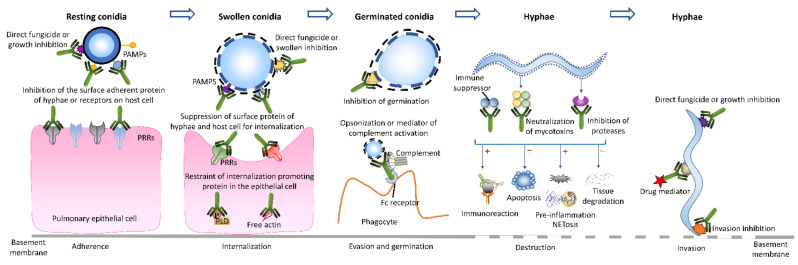
Monoclonal antibody-based modes to suppress or eliminate *Aspergillus*.

Invasive aspergillosis is associated with a complex process of adherence, internalization, and invasion of *Aspergillus* and the involvement of various proteases and toxins. Thus, mAbs specific to those molecules protect the host and aid removal of *Aspergillus* pathogens by suppression of adherence [109,110,111], opsonization [112], neutralization of toxins or enzymes [113], complement activation and directing fungicidal activity [110,114,115] (Figure 2). For example, the mAbs binding to sialic acid [27] and galactosaminogalactan [35] on *Aspergillus* or to epithelia receptors, such as fibronectin [27,35] or E-cadherin [116,117], may interfere with *Aspergillus* adhesion, endocytosis and agglutination and downregulate the pathogenicity of the fungi. *Aspergillus* toxin neutralization restricts epithelial internalization and promotes the immune response to the pathogens, thus reducing invasion and dissemination of *Aspergillus* [33,43]. All of these approaches have promise in protecting the host from fungal attack. Moreover, some mAbs have catalytic ability that can hydrolyse the target antigens and directly destroy the pathogens, independently from the individual’s immune system [118]. Additionally, the mAb can be used as a mediator to direct the anti-fungal drug to the infection site, enhancing the anti-fungal activity.

### 4.3. Monoclonal Antibody-Based Immunotherapeutic Modes for Invasive Aspergillosis

The number of mAb-related therapeutic strategies investigated for IA is steadily expanding and includes both in vitro and in vivo studies, although the immune treatment of IA is still in its infancy. The mAb-guided therapeutic regimens have gained widespread interest in IA treatment because mAbs bind to the specific antigens and can target the *Aspergillus* infection quickly and accurately. In 1993, Frosco et al. generated five mAbs (BB11, MB8, KD5, GD11, and CCIII 19) against elastase that effectively inhibited the enzymatic activity of elastase in vivo, but found that these mAbs did not protect immunocompromised mice from fatal *A. fumigatus* infection. This suggested elastase may not be the principal virulence factor included in IPA mice [119]. Since then, further mAbs directed against a large variety of cell wall antigens of *A fumigatus* have been reported, and antifungal activity tested both in vitro and in vivo. All of the mAbs related to the treatment of *Aspergillus* infection are listed in Table 2, including the therapeutic effects, antibody subclass and references. 

As shown in Table 2, various mAbs can act as an anti-fungal agents and protect the hosts directly in different ways based on the binding between the antibody and the antigen. In addition, those mAbs specific to receptors such as Axl that have inhibitory effects on specific parts of the immune response may improve the outcome of IA in a mouse model [124]. MAbs can also be used as a carrier to direct antifungal drugs to the diseased region, resulting in precise treatment with fewer side effects. For instance, alliinase conjugated mAb produces cytotoxic allicin molecules in the presence of alliin and effectively killed the fungi in vitro [127]. This study opens a new door to treat serious *Aspergillus* infection using antibody-directed enzyme prodrug technology (ADEPT) [128], which can guide the enzyme to activate a prodrug at a specific infection site, thus diminishing the adverse effect to other parts of the body.

Additionally, some mAbs that do not have fungicidal properties could have antifungal effects by conjugating with a radiation emitter. Known as radioimmunotherapy [126], this has been widely utilized in anti-cancer treatment and shows very promising results [129]. Although it has yet to be explored with IA, radioimmunotherapy showed fungicidal activity against *Cryptococcus neoformans* in vitro by significantly decreasing the tissue burdens of cryptococcosis in a mouse model, as well as extending the survival time with minimal toxicity [130]. 

Taken together, the mAb-mediated immune therapy strategies are currently associated with the direct interference of pathogen attachment, inhibition of conidia germination, restraint of hyphal growth, fungal growth inhibition or fungicidal activity, fungal protease inhibition to prevent the tissue digression, immune activity enhancement of the host and drug mediation (Figure 2). Thereby, this new immune treatment avenue hinders the fungal growth and exerts a protective effect to the animal in vivo. In addition, mAbs can take part in the drug transportation, leading to specific and precise mAb-mediated treatments with less side effects. These pre-clinical studies demonstrate an extremely promising future in immune treatment of IA. However, most studies focus on the final therapeutic effects in the in vitro or in vivo experiments, but rarely explore the detailed fungicidal mechanisms of mAbs in IA treatment. Further studies are required to illustrate these specific mechanisms.

## 5. Perspectives and Conclusions

Mab-mediated immuno-diagnostic and -therapeutic approaches have potential to improve the outcome of IA. However, further studies are needed to realise this promise in a clinical setting where there is high IA morbidity and morbidity. Novel immune-molecular imaging modalities offer the promise of a rapid test that can identify *Aspergillus* species in vivo, offsetting the need for invasive and potentially harmful tests, such as biopsies or BALF, in severely neutropenic or thrombocytopenic patients. Linking mAbs to differing contrast agents may allow their use with several imaging platforms such as PET/MRI. Secondly, given that IA patients have high mortality, other novel strategies for preventing *Aspergillus* infection are needed. Passive immunisation with mAbs against *Aspergillus* species appears to improve survival in mouse models of IA, but there are no reports of humanising mAbs or the potential for use as prophylaxis in humans or as treatment to augment the activity of antifungal pharmaceutical agents.

The clinical diagnosis and management of IA remains challenging, because of the non-specific symptoms coupled with the lack of precise and non-invasive diagnostic methods. The delayed diagnosis leads to delayed antifungal treatment, aggravating the disease progression. The mAb-mediated diagnostic methods with high sensitivity and specificity, including molecular imaging, offer the promise of accurate diagnostic modalities that limit this vicious cycle of progressive infection by early detection of the fungal wall antigens or secreted molecules. In addition, the pathogen-specific mAb-directed antifungal regimens show therapeutic promise in IA by inhibiting important steps in the pathogenesis of IA, enhancing the host response and directing fungicidal activity. In conclusion, the mAb-based diagnostic and therapeutic strategies have been widely applied in modern medicine, and further innovation can be expected to improve the diagnosis and treatment of IA. However, as there are many challenges in designing, powering and conducting clinical trials with relevant endpoints, progress has been slow. All-cause mortality at day 42 is useful for acute pulmonary IA but does not work well for slowly progressive infection. The EORTC-MSG defined an overall response endpoint that requires improvement in all three sub-elements (clinical, radiological and mycological) so that if lesions are stable, the response is defined as failure [131]. Despite these difficulties, there is recognition of an unmet need [132]. At present, most of these antibody-guided diagnostic and therapeutic investigations are at the pre-clinical stage, and multi-centre collaboration offers the opportunity to translate these promising agents into clinical practice [133].

## Figures and Tables

**Table 1:** Advantages and disadvantages of classical diagnostic methods for IA.

Diagnostic Methods	Advantages	Disadvantages
Histopathology and microbiology	Gold standard Pathologic changes of the tissue Morphology of the *Aspergillus*	Invasive operation High requirements for specimen quality Technology dependent on the technician Time-consuming False-negative
Fungal biomarker assay	Early detection Non-invasive Various sample resources Platform widely available Rapid turnaround time	False-positive False-negative Unknown pathogen species Unknown infection site
*Aspergillus* polymerase chain reaction (PCR) test	Specific species Various sample resources Rapid turnaround time	Lack of standardization Contamination can be problematic
CT scanning	Fast and non-invasive Location of infection site Lesion size and number	Non-specific Pathogen not identified Viability of pathogen not indicated
Serological antibody test	Easily performed on readily accessible samples	False-negative in immunocompromised host

**Table 2:** Monoclonal antibody-mediated therapeutic effects in *Aspergillus* infection.

Therapeutic Effects	MAb	Subclass	References
Fungal growth inhibition/fungicidal activity (in vitro)	C7, K10, A9, Mab-7, SMB19, R-5, MS112-IIB1, YW327.6S2, 3G11 and 5H5	IgM, IgG, IgG1, IgG3	[110,112,113,115,120,121,122,123,124]
Fungal growth inhibition/fungicidal activity (in vivo)	K10, A9, 2G8, R-5, 3G11 and 5H5	IgM, IgG1, IgG2b, IgG3	[112,115,118,123,125,126]
Germination suppression (in vitro)	K10, A9, 2G8, R-5, 3G11 and 5H5	IgM, IgG1, IgG2b, IgG3	[112,115,118,123,125,126]
Attachment inhibition (in vitro)	2G8, Mab-7, AK-14	IgG2b, IgM	[109,110,111,125]
Protease inhibition (in vivo)	BB11, MB8, KD5, GD11, and CCIII 19	IgG1 and IgG2a	[119]
Immunological enhancement (in vitro and in vivo)	A9, SMB19, MS112-IIB1, 3G11, 5H5, YW327.6S2	IgG1, IgM, IgG1, IgG3, IgG	[112,113,122,123,124]
Drug mediator (in vitro and in vivo)	MPS5.44	IgM	[127]

## Data Availability

Not applicable.

## Abbreviations

IA	invasive aspergillosis
mAbs	monoclonal antibodies
mAb	monoclonal antibody
HSCT	allogeneic hematopoietic stem cell transplant
SOT	solid organ transplant
PAMPs	pathogen-associated molecular patterns
PRRs	pattern recognition receptors
Cal A	calcineurin A
GAG	galactosaminogalactan
NETs	neutrophil extracellular traps
ROS	reactive oxygen species
PLD	phospholipase D
EORTC/MSGERC	European Organization for Research and Treatment of Cancer and the Mycoses Study Group Education and Research Consortium
PCR	polymerase chain reaction
GM	galactomannan
BALF	bronchoalveolar lavage fluid
LFD	lateral flow device
IHC	immunohistochemistry
IF	immunofluorescence
PET	positron emission tomography
SPECT	single-photon emission computed tomography
BLI	bioluminescence imaging
LSFM	light sheet fluorescence microscopy
ADEPT	antibody-directed enzyme prodrug technology
FDA	Food and Drug Administration.
